# The Prospect of Repurposing Immunomodulatory Drugs for Adjunctive Chemotherapy against Tuberculosis: A Critical Review

**DOI:** 10.3390/antibiotics10010091

**Published:** 2021-01-19

**Authors:** Chiyun Lee, Sanjib Bhakta

**Affiliations:** 1 Department of Biochemistry, University of Cambridge, Hopkins Building, Tennis Court Road, Cambridge CB2 1QW, UK; cl724@cam.ac.uk; 2 Mycobacteria Research Laboratory, Department of Biological Sciences, The Institute of Structural and Molecular Biology, Birkbeck, University of London, Malet Street, London WC1E 7HX, UK

**Keywords:** tuberculosis, drug resistance, repurposing, immunomodulatory drugs, NSAIDs, adjunctive therapy, potentiator

## Abstract

Tuberculosis (TB) remains a global health emergency, with an estimated 2 billion people infected across the world, and 1.4 million people dying to this disease every year. Many aspects of the causative agent, *Mycobacterium tuberculosis*, make this disease difficult for healthcare and laboratory researchers to fight against, such as unique pathophysiology, latent infection and long and complex treatment regimens, thus causing patient non-compliance with the treatment. Development of new drugs is critical for tackling these problems. Repurposing drugs is a promising strategy for generating an effective drug treatment whilst circumventing many of the challenges of conventional drug development. In this regard, the incorporation of immunomodulatory drugs into the standard regimen to potentiate frontline drugs is found to be highly appealing. Drugs of diverse chemical classes and drug categories are increasingly being evidenced to possess antitubercular activity, both in vitro and in vivo. This article explores and discusses the molecular entities that have shown promise in being repurposed for use in anti-TB adjunctive therapy and aims to provide the most up-to-date picture of their progress.

## 1. Tuberculosis: The Disease, the Immunobiology and the Available Treatment

Despite the recent resurgence in political will to fight tuberculosis (TB), the disease remains as a leading cause of death from a single infectious agent around the world [1]. Every year, an estimated 1.2 million deaths are caused by TB in the HIV-negative population with a further 208,000 deaths from those who are HIV-positive. Roughly a quarter of the world is infected—around 2 billion—and 10 million new cases are said to develop every year, making TB still an extremely prevalent disease. Moreover, the disease has a distinct geographical distribution, with the most heavily burdened countries often being of lower income, strongly coinciding with where HIV infection rates are rampant too. The milestones and targets set by The End TB Strategy push public healthcare and interdisciplinary science capabilities to the limit. 

The causative agent of TB is *Mycobacterium tuberculosis*, a slow-growing acid-fast bacillus manifesting itself primarily as a respiratory pathogen causing pulmonary disease. It can also cause extra-pulmonary TB via systemic infection, and its pathogens are capable of crossing the blood–brain barrier. This dangerous pathogen is required to be handled in a Biosafety Level 3 environment, and in conjunction with its slow-growing nature, makes for an especially difficult bacterium to study. Adding to its unique physiology are its cell wall [2], biofilms [3] and notorious drug efflux pumps [4], which work together to efficiently ruin many of our attempts to develop drugs against it. 

Granulomas are the iconic immunological host response to TB infection [5]. The structurally organised cluster of macrophages and lymphocytes is the immune system’s most fundamental strategy for containing the spread of infection and eliminating the entrapped bacilli [6]. Although granulomas are capable of sterilising infection foci, *M. tuberculosis* can enter a state of dormancy in response to stresses making it remarkably more tolerant to immune responses as well as antibiotic chemotherapy [6]. The majority of the infected population is described to be in this state of latent TB infection (LTBI), showing no overt symptoms. 

Reawakening from this persistence state is a slow, stochastic and poorly understood process. TNF-α is a linchpin of the anti-TB response as it recruits immune cells and drives the formation and maintenance of granulomas. Transient immunosuppression, for example in anti-TNF therapy, provides stimulus for bacilli to reawaken and cause active disease [7,8]. Immunosuppressive diseases, such as diabetes mellitus and HIV, are linked with higher susceptibility to active TB. As much as 10% of the TB-infected population in HIV endemic regions are estimated to have active disease as a result of co-infection [1].

The nature of TB enhances the development of resistance to antibiotics. Bacilli emerging from persistence generate a constant influx of cells of varying levels of physiological resistance, facilitating the selection of genetic resistance. Granulomas also diminish the penetration of antibiotics, exposing the bacilli to a decreased concentration of the drugs too [9]. Finally, it is no surprise that the current gruelling 6-month regimen of rifampicin, isoniazid, ethambutol and pyrazinamide sees suboptimal compliance, further promoting resistance. 

The prospect of vaccines eradicating TB seems far into the future as the latest M72/AS01E vaccine only provides protection in half of those administered after three years [10], and efficacy of the BCG vaccine against adult pulmonary disease is highly disputed [11]. Moreover, it took an astonishing 40 years, after the golden era of antibiotic discovery, for an anti-TB drug with a completely unique mechanism of action to be developed and then become approved by the FDA [12,13]. It is evident that the low-hanging fruit for novel antimicrobials has been long picked.

## 2. The Prospect of Repurposing

In the midst of all these interconnected factors that hinder TB eradication, drug repurposing is a prospective strategy. Drugs that are repurposed for TB can fast-track initial stages of drug development as safety trials, and pharmacokinetic/pharmacodynamic (PK/PD) testing has been performed during testing for other disease conditions. The potential reduced financial investments and faster rollouts of novel treatment strategies make repurposing highly desirable in the light of the new drug development pipeline drying up. There are numerous leaps forward of repurposing in other fields, as well as many prospective leads in the repurposing of anti-infectives for TB, such as anti-helminthic drug pyrvinium pamoate, which protects against extensively drug-resistant TB (XDR-TB) in murine models [14], and various antibiotic classes, including fluoroquinolones, nitroimidazoles, β-lactam/β-lactamase inhibitor combinations and oxazolidinones. Moxifloxacin has been successfully repurposed as second-line treatment for TB [15], and several other fluoroquinolones are in clinical trials to shorten TB treatment or are in pre-clinical development [16], whilst novel derivatives from nitroimidazole, pretomanid and delamanid have been approved for use in TB patients and have numerous late clinical trials aiming to incorporate them into combination therapies [17]. There has also been a resurgence of interest in β-lactams owing to the discovery of β-lactamase inhibitors, clavulanate and avibactam, and they are featured in several clinical trials [18,19,20,21]. Although originally for Gram-positive bacteria, oxazolidinones—linezolid and its derivatives—are in many clinical trials, with linezolid already approved for use in TB [22]. In addition, the antiparasitic artemisinin is currently receiving a lot of attention from the research community for its ability to disrupt signalling that regulates persistence in *M. tuberculosis* [23,24,25,26]. 

In a disease with such a heavy involvement of immunology as TB infection, the potential for host-directed therapies (HDTs) is overwhelming [27,28]. The power to reduce excessive lung damage and enhance bactericidal capacity of the immune system is immense, all the while avoiding the risk of resistance seen in antimicrobials. A controlled dampening of the immune system to stimulate reawakening of the bacteria may be exploited to predispose the awakening bacteria to elimination by first-line drugs and shorten treatment due to elimination of the dormant reservoir [29]. 

Whether repurposing immunomodulatory drugs is a selective leg-up or a silver bullet will require further investigation and close analysis of our first steps in this approach. 

## 3. Repurposing Immunomodulatory Compounds

There are numerous aspects of inflammation that differentially determine the outcome of immunity against TB. Excessive inflammation and necrosis can overwhelm the lung’s ability to repair itself and even spread the infection, whilst too weak of an immune response will disrupt granuloma integrity and release the bacilli [30]. Little has been elucidated about what predisposes an infected individual to either extreme of the inflammatory response, but in the meantime, there have been countless attempts to pharmacologically modulate and correct the immune response in adjunct therapy alongside the standard anti-TB drug regimen (see Table 1 for a general overview). 

One of the most investigated out of these is vitamin D due to the link between vitamin D deficiency and TB susceptibility and its potential to stimulate autophagy and the production of antimicrobial peptides [31]. Another welcome observation of vitamin D is its direct growth inhibition of mycobacteria in vitro [32]. Being essential for our natural consumption, vitamins are particularly easy to investigate in clinical trials as they come with few safety concerns, attributed to the numerous studies on vitamins in host-directed therapy and countless retrospective studies on vitamin D supplementation [33]. However, studies show conflicting results on the efficacy of vitamin D in adjunct therapy, with some showing accelerated sputum culture conversion only in multi-drug-resistant TB (MDR-TB) patients but not overall in the study [34], whilst others detect no effect whatsoever [35]. Many studies do highlight an improved quality of life for a specific subset of patients, which perhaps points to us requiring more investigation on patient markers for effective vitamin D adjunctive therapy, the sensitivity for which retrospective studies are likely to lack. Despite so much investigation in this area, the future of vitamins in TB is still unclear. Regardless, it is tempting to make vitamin supplementation a standard procedure for TB-infected individuals in regions of the world where malnutrition and natural vitamin deficiencies suppresses the immune system. 

Corticosteroids are a clinically important drug class used in modulating the immune system in other illnesses and have also attracted a lot of attention for potential host-directed therapy. The more pronounced differential organ-specific effects of corticosteroids may make it hard to repurpose them for use in a general immunomodulatory adjunct therapy, however [36]. This leaves corticosteroids to be primarily used for reducing excessive inflammation in the severest cases of meningitis TB for now, as a study of prednisolone in HIV-associated pulmonary TB showed no survival benefit but showed a reduction in clinical complications [36,37]. Their ability to influence anti-TB drug accumulation in different compartments of the body can perhaps be exploited in a customised organ-specific therapy for various extrapulmonary foci of TB infection.

### 3.1. Immunomodulation Is Diverse

Immunomodulatory properties are not exclusive to drugs classically considered as general-purpose anti-inflammatory drugs. The histone deacetylase inhibitor, phenylbutyrate, used originally for urea cycle disorders, has seen several successful clinical trials that indicate a diverse array of immunomodulatory functions in TB infection [38,39,40]. Furthermore, the notorious thalidomide, used originally for morning sickness, has been frequently investigated for its use in meningitis TB therapy due to its antagonistic effect on TNF-α for its potential to limit excessive inflammation [41,42]. Though alleviating symptoms, the counter-productive T cell co-stimulatory properties of thalidomide and, most importantly, its teratogenicity restrict its translation of immunomodulatory properties in the clinic [43]. 

The chemical diversity of molecular entities possessing immunomodulatory potential repeatedly exceeds expectation. Auranofin is a peculiar molecule consisting of a gold atom conjugated to a saccharide, used in the treatment of rheumatoid arthritis, which can make *M. tuberculosis* prone to reactive oxygen species (ROS) and reactive nitrogen intermediates (RNI) attack, currently investigated in a phase II trial (NCT02968927). The concept of repurposing drugs opens our eyes to the opportunity to make better use of our entire arsenal of therapeutic molecules. Our conventional expectation for what confers therapeutic efficacy may be limiting our potential to treat disease. 

A wilder example is seen in doxycycline. Although originally developed for its effect on bacteria and not the host, doxycycline in fact exerts a direct effect on human matrix metalloproteinases. *M. tuberculosis* infection reprograms the host tissue environment and causes destruction to lung tissue, making the potential of doxycycline to counteract these phenomena highly attractive [44]. A mouse model of TB infection demonstrates the potency of doxycycline in stabilising lung tissue integrity and increasing the concentration of first-line anti-TB drugs in the lung tissue [45]. A clinical trial investigating its efficacy in humans has been completed, and its results are awaited (NCT02774993). 

A similar effect of optimising drug distribution is seen in drugs used for cardiovascular disorders. Verapamil, a drug used for treating high blood pressure, potentiates standard TB therapy in mice, additionally exerting a direct inhibitory effect on *M. tuberculosis* [46,47]. Its ability to reduce the MIC of bedaquiline in vitro and increase the bioavailability of bedaquiline in mouse models makes it a very promising drug to follow in the future [48].

The cholesterol-lowering statins show a variety of properties which give them good potential for repurposing in adjunctive TB therapy. The effect of pravastatin on macrophages phenocopies that of classically activated macrophages, whilst all members of the drug class exert an inhibitory effect on TB-infected macrophage ex vivo models [49]. Some statins also show synergy with first-line drugs in vitro and in vivo, highlighting their multi-pronged effect in inhibiting TB growth. A retrospective analysis reveals protection from LTBI in statin users [50], and one phase II trial investigating rosuvastatin in adjunctive therapy is underway (NCT04504851). However, concerns about the detrimental effects on host immunity arise from cases of rapid progression of bladder cancer upon statin use [51], as well as the drug interaction with the anti-TB drug rifampicin [44], warranting further investigation before repurposing is possible [52]. 

The same study by Magee, M.J. et al. [50] revealed metformin use to also protect against LTBI, once again highlighting the well-established link between diabetes mellitus and TB susceptibility [53]. Following from these disease associations, the evidence supporting metformin use as an adjunctive drug is building in both mouse models [54] and in the clinic [55,56]. 

Another large class of drugs being investigated for anti-TB adjunct therapy is the phosphodiesterase inhibitors (PDE-I). Unlike non-steroidal anti-inflammatory drugs (NSAIDs), phosphodiesterase inhibitors target a family of host enzymes that are divergent in function. Although PDE-I all block the degradation of second messenger intracellular cyclic nucleotides to modulate intracellular signalling, the distributions of the different phosphodiesterases is tissue-specific, and thus inhibitors of different isoforms of phosphodiesterase produce a unique profile of effect. In one experimental model the PDE-5 inhibitor sildenafil is observed to prolong death in mice without decreasing bacterial burden, whilst PDE-3 inhibitor cilostazol does in fact reduce bacterial load in lungs [57]. This can be somewhat explained by how PDE-5 is found predominantly in lung tissue and PDE-3 in macrophages and platelets [58]. In drugs with such varied modulatory effects on intracellular signalling, it would be important to commence animal model studies promptly to evaluate the net effect of all the individual effects on different tissues. Overall, various inhibitors of PDE-3, -4 and -5 have demonstrated excellent anti-TB activity and synergy with frontline drugs in mouse and rabbit models and are soon to enter clinical trials as adjunct therapy [59,60,61]. 

### 3.2. Anti-Cancer Drugs

Repurposing for TB therapeutics is recently taking inspiration from cancer therapeutics, which perhaps is no surprise due to the role of immunomodulation at the microenvironment level in both diseases. Imatinib targets host tyrosine kinases and was originally designed for Abl kinase-driven cancers, though mouse model experiments of imatinib against TB show that its immunomodulatory effects are translatable [62]. Indeed, a recent case was of a chronic myeloid leukaemia patient undergoing imatinib therapy whose latent TB had reawakened [63]. A phase II trial is scheduled to commence shortly to investigate this (NCT03891901). TNF-α inhibitors and LT-α inhibitors, highly successful in cancer treatment, also show potential for repurposing, as an in vitro granuloma model reveals their ability to resuscitate dormant TB via controlled immunosuppression [64]. There is a recent burst of exploration into similar molecules, such as nilotinib, gefitinib and fostamatinib, each showing varied mechanisms of action, for example, enhancing autophagy [65,66,67].

Cancer research also arguably boasts the most innovative strategies in therapeutics. Biologics contrast the small molecule drugs discussed thus far but have demonstrated many successes in cancer. Denileukin diftitox, used against T cell lymphomas, is a fusion protein of diphtheria toxin and human CD25 receptor, targeting regulatory T cells for killing by the toxin component to revert immunosuppressive microenvironments in a mouse model [68]. Research here is still in its early stages, and it has been proposed that these biologics may in fact be detrimental in TB infection [69]. The work needed for successful repurposing is immense, but these early findings on the potential of biologics in anti-TB therapeutics may breathe new life into how we approach developing new therapies against the disease. Chimeric antigen receptor (CAR)-T cell therapy describes the ex vivo gene editing of antigen receptors of patient T cells before re-introduction into patients for enhanced immunity in cancers [70]. Perhaps CAR-T cell therapy can be adopted for use in the treatment of TB. 

There remain many drugs of diverse purpose that are investigated for their repurposing value in isolated pockets of research. Diosmin, used normally for haemorrhoids, was discovered in silico as a repurposable drug and demonstrates protection in a Drosophila TB model, also highlighting the potential of in silico methods to find new leads [71]. Lipoxygenase inhibitors were proposed as immunomodulators after inhibition of host lipoxygenase improved survival in a mouse model [33,72]. Antiviral isoprinosine resuscitates the immune system dampened by viruses during infection and can potentially be repurposed for use against TB [73]. In addition to phenylbutyrate, histone acetylase inhibitors valproic acid and vorinostat, originally developed for neurological disorders and cancers, respectively, show synergy with frontline drugs in a macrophage model [39]. Phenothiazines have been deemed too toxic in several clinical trials [74,75], whereas the MIC of the thiocarbamate, disulfuram, was found to be too high. Disulfuram has recently been further repurposed to act as a copper ion chelator to deliver bactericidal activity in vitro [76]. 

### 3.3. Non-Steroidal Anti-Inflammatory Drugs

Non-steroidal anti-inflammatory drugs (NSAIDs) are a large and diverse class of drugs that inhibit cyclooxygenase (COX) to reduce prostaglandin production, thereby alleviating inflammation, fever and pain [77]. Although the WHO recommends administering NSAIDs for TB patients, they are aimed at relieving the joint pain caused by anti-TB drugs and directly helping with the infection itself. In recent years, there has been a growing body of science to support the use of NSAIDs in adjunctive therapy, especially as some members of the NSAIDs chemical class are available as over-the-counter medication, making them easier to repurpose. 

The most studied NSAID currently in TB research is ibuprofen. This common drug found in most households has been found to demonstrate a direct inhibitory effect in whole-cell screening assays [78] and most importantly provide protection against TB in mouse animal models [79,80]. A newer paper calls into question the validity of the disease model in the latter papers, however, criticising the use of intravenous (IV) infection as opposed to a more representative respiratory infection route used in their paper, demonstrating that ibuprofen and celecoxib are in fact detrimental for immunity against TB [81]. The author reasoned that an IV infection using an unphysiological thousand-fold higher dose of *M. tuberculosis* would trigger massive inflammation which an anti-inflammatory drug would, of course, alleviate. The importance of the quality of disease models is acutely emphasised in a complex disease like TB. Nevertheless, we await the results for a phase II clinical trial investigating the use of ibuprofen as adjunctive therapy in extensively drug-resistant TB (NCT02781909). The results from this study will be valuable in discerning the significance of the many conflicting findings of the pre-clinical research. 

Aspirin is another common household NSAID which received attention in the TB research community. Although initially shown to potentiate the first-line drug, pyrazinamide, in a murine model [82], its undesirable drug interactions with isoniazid [83] and lack of significant effect observed in the more powerful rabbit TB model shows little promise for aspirin as an immune-modulatory adjunctive drug [43]. Instead, aspirin holds greater potential for use as a general anti-inflammatory drug for a disease driven by excessive inflammation, such as meningitis TB, as opposed to one which demands a finer balance of the different arms of immunity as in pulmonary TB. One clinical study showed aspirin in combination with corticosteroids reduces strokes and mortality in tuberculous meningitis [84], and we will see the results of a similar ongoing study soon (NCT02237365). 

Etoricoxib, meloxicam and celecoxib form the remaining NSAIDs which have been studied in clinical trials. The results from a recent study attempting to determine the safety of etoricoxib as an adjunct for the novel H56:IC31 vaccine are eagerly being awaited as we see the first steps of a vaccine that targets reinfection and relapsing TB infection, as well as a NSAID used as an immunostimulant for vaccine response (NCT02503839). The results from the long-completed phase III clinical study on meloxicam in preventing TB-immune reconstituted inflammatory syndrome (TB-IRIS) have yet to be published (NCT02060006). Finally, celecoxib was investigated in a phase I ex vivo trial for adjunctive therapy which showed no effect, though the significance of an ex vivo model is unknown (NCT02602509).

Some NSAIDs which have fallen away from the spotlight are oxyphenbutazone, diflunisal and bromfenac [85]. Their progression beyond their in vitro inhibitory potential is thwarted perhaps by concerns about toxicity [86]. The toxicity profile of diclofenac appears to be deemed too dangerous for its observed synergy in a murine model with former first-line antibiotic, streptomycin, to warrant further investigation [87].

The host-directed therapeutic effects of NSAIDs vary predominantly via their different pharmacokinetic properties, as opposed to varying via alternative mechanisms, exerting differential effects based on tissue location and cell type. Carprofen is an NSAID recently found to lean strongly towards direct antitubercular-specific mechanisms of action, in contrast to the majority of NSAIDs, boasting a minimum inhibitory concentration (MIC) of 40 µg/mL, well within the MIC range of most antibiotics [78]. Traditionally, a drug with a pathogen-directed activity would bring up concerns about the development of resistance. However, carprofen demonstrates a pleiotropic mechanism of action, disrupting efflux pumps, biofilms and membrane potentials, which lessens the risk and effect of resistance mutations [88]. In conjunction with its classical NSAID effects, carprofen proves to be a promising candidate for further testing in animal models for its potential to simultaneously revert tolerance to first-line TB drugs through inhibition of efflux pumps, directly inhibit growth and exert a host-directed immunomodulatory effect. Having been used in human medicine for over 10 years but later repositioned for veterinary use for commercial reasons, carprofen should face greater ease in repurposing as an anti-TB adjunct drug [89]. 

The discovery of more anti-tuberculosis adjunctive drugs and potentiator molecules may be facilitated by following strategies used in the development of an effective combination therapy against TB. Hypomorphs describe *M. tuberculosis* strains engineered to have reduced expression of a gene that is essential for function. Screening a library of repurposable compounds against various hypomorph strains allows for greater sensitivity in detecting compounds with inhibitory effects on particular pathways [90]. This may be especially important in targeting pathways that are more difficult to observe as having relevance during initial in vitro whole-cell screens but are critical in vivo, such as mycobacterial HsaD. The *hsaD* (Rv3569c) gene is found to be essential for mycobacterial cholesterol metabolism within macrophages, and its hypomorphs have been used to identify and develop novel chemical leads in antibiotic discovery [91], highlighting the potential for a similar approach to be used to identify candidate repurposed drugs. 

As for the more classical NSAIDs, given the role of prostaglandins in antagonising TNF-α, IL-1β, ROS and RNI, there is obvious potential for NSAIDs enhancing killing of *M. tuberculosis* through inhibition of COX [77]. However, the whole picture seems far more complex as prostaglandins are also essential for reducing early mitochondrial damage and bias macrophages towards apoptosis instead of necrosis [92]. As apoptosis drives both innate and adaptive immune defence mechanisms, prostaglandins have effects on both arms of immunity. Furthermore, some virulent strains of TB can be seen to inhibit COX-2 production themselves [77]. In the study of such multi-functional drugs against such a multi-faceted disease, it is critical to use animal models that more closely mimic the intricacies of human pulmonary TB like cavitating lung granulomas, which the rabbit or C3HeB/FeJ mouse models achieve, as this would allow the potential of NSAIDs to be assessed with greater accuracy. Additionally, while NSAIDs have a justified role due to their antimicrobial mechanisms of action in the proposed adjunctive therapy, the timing of administration and route of drug delivery would be critical in modulating the immune response appropriately. Such resource-intensive testing for whether individual TB patients are appropriate for administration strays far from a simple and standardised drug regimen for reducing the prevalence of such a widespread disease. Only further research and clinical testing can help us answer these questions. 

**Table 1:** Summary table of the most promising immunomodulatory drugs discussed in this literature review currently under investigation for their potential to be repurposed as an adjunct drug for the standard anti-TB drug regimen.

Drug Class	Name	Original Indication	Delivery Route	FDA Approval	Clinical Trial Progression	Availability	Key References
Biologic	Denileukin diftitox	Cutaneous T cell lymphoma	IV	1999	--	PO	[68]
CCB	Verapamil	Angina	Oral	1981	--	PO	[46,47]
HDACi	Phenylbutyrate	Urea cycle disorders	Oral	1996	NCT01580007 Phase II trial completed 2018	PO	[38,39,40]
HDACi	Valproic acid	Epilepsy	Oral	1978	--	PO	[39]
HDACi	Vorinostat	Cutaneous T cell lymphoma	Oral	2006	--	PO	[39]
LOi	--	Asthma, neoplasms, arthritis	--	--	--	--	[33,72]
NSAID	Aspirin	Arthritis, analgesic	Oral	1950	NCT02237365 Phase II trial awaiting results	OTC	[43,82,83,84]
NSAID	Carprofen	Analgesic	Oral	1987	--	VUO	[78,88]
NSAID	Celecoxib	Arthritis	Oral	1998	NCT02602509 Phase I trial completed	PO	[33,81]
NSAID	Etoricoxib	Inflammatory disorders	Oral	--	NCT02503839 Phase I trial underway	PO	[29,33]
NSAID	Ibuprofen	Arthritis, analgesic	Oral	1974	NCT02781909 Phase II trial underway	OTC	[79,80,81,82]
NSAID	Meloxicam	Arthritis	Oral	2000	NCT02060006 Phase III trial awaiting results	PO	[33]
PDEi	Cilostazol	Cardiovascular disorders	Oral	1999	--	PO	[57,58,59,60,61]
PDEi	Sildenafil	Erectile dysfunction	Oral	1998	--	PO	[57,58,59,60,61]
Statins	Pravastatin	Cardiovascular disorders	Oral	1991	NCT04504851 Phase II trial underway	PO	[49]
Statins	Rosuvastatin	Cardiovascular disorders	Oral	2003	NCT04504851 Phase II trial in planning	PO	[50]
TC	Doxycycline	Bacterial infections	Oral	1967	NCT02774993 Phase II trial awaiting results	PO	[44,45]
TNFi	--	Autoimmune disorders	--	--	--	--	[8]
TKI	Fostamatinib	Chronic immune thrombocytopenia	Oral	2018	--	PO	[65,66,67]
TKI	Gefitinib	Metastatic non-small cell lung cancer	Oral	2003	--	PO	[65,66,67]
TKI	Imatinib	Chronic myeloid leukaemia	Oral	2001	NCT03891901 Phase II trial in planning	PO	[62,63]
TKI	Nilotinib	Chronic myeloid leukaemia	Oral	2007	--	PO	[65,66,67]
Vitamin	Vitamin D	Vitamin	Oral	--	Several clinical trials completed	OTC	[31,32,33,34,35]
--	Auranofin	Rheumatoid arthritis	Oral	1985	NCT02968927 Phase II trial awaiting results	PO	[93]
--	Diosmin	Haemorrhoids	Oral ^1^	--	--	--	[71]
--	Isoprinosine	Viral infections	Oral	--	--	--	[73]
--	Thalidomide	Leprosy	Oral	1998	Several clinical trials completed	PO	[38,39,40]

Abbreviations: CCB, calcium channel blocker; HDACi, histone deacetylase inhibitor; IV, intravenous; LOi, lipoxygenase inhibitor; NSAID, non-steroidal anti-inflammatory drug; OTC, over the counter; PDEi, phosphodiesterase inhibitor; PO, prescription-only; TC, tetracycline; TKI, tyrosine kinase inhibitor; TNFi, tumour necrosis factor inhibitor; VUO, veterinary use only. ^1^ Also available as topical medication.

## 4. Conclusions

**Figure 1:**
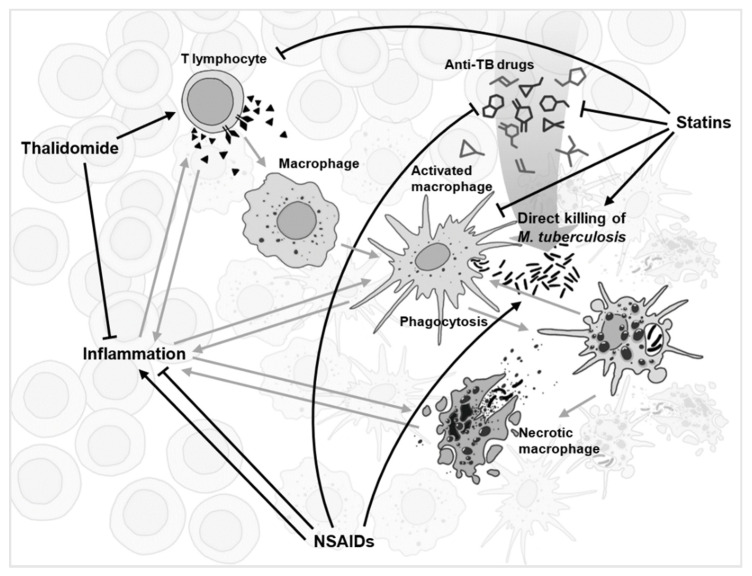
A simplified schematic of the complex network of interactions (grey) during tuberculosis infection within a granuloma. Several drugs with the most conflicting experimental evidence, and yet with repurposing potential for anti-TB adjunct therapy, are displayed with their suggested network of interactions shown (black).

In the midst of rising antibiotic resistance and the uncertain future for conventional new drug design, repurposing immunomodulatory drugs has potential to hold tremendous value for TB research. Despite promising clinical trials in this area, the many conflicting pieces of evidence leave some key questions unanswered (Figure 1). Are the effects of immunomodulatory drugs suitable for incorporation into a standardised anti-TB treatment or should they be reserved for patient-tailored treatment? Can drugs be directly repurposed against TB or will drugs have to undergo further chemical modification, thereby reducing/increasing further challenges? How much of the effect of these repurposed drugs is host-directed and how much is pathogen-directed? Following this, what are the long-term off-target/adverse effects on the host and how great is the risk of development of resistance? Many inquiries remain before repurposed immunomodulatory drugs can become a standard anti-TB chemotherapy. To tackle the questions that hinder us from achieving this goal, multi-disciplinary research must be conducted at a greater pace and scale, and yet still with caution, given the significant potential of immunomodulation to also cause complexity in TB pathophysiology.
